# Rheumatism

**Published:** 1875-01

**Authors:** 


RHEUMATISM.—It is entertainingly related that John Trotman of Wathena, Mo., was a great sufferer from this incorrigible infirmity. Physicians were in vain, and were likely to be, inasmuch as the complaint was rheumatism and the patient was 60 years old. So Mr. Trotman, in the absence of his wife, tied two flat-irons under his chin and (in some unaccountable way) his hands behind his back, and thus accoutered plunged into Peter's Creek, where he was found soon after quite free from rheumatism and from everything else except the flat-irons. The man who was hired to watch Mr. Trotman, and who didn't watch him, hasn't been arrested, though he should be, and at least fined for mitigated manslaughter. The kind of rheumatism which they have in Connecticut doesn't seem to affect the mind, but it is very steady and lasting. A doctor was called in East Hartford last week to a man (aged 66) who was excessively rheumatical. "How long have you had it?" says the doctor. "Forty years," responded the sufferer. The doctor left without prescribing. We cannot tell how much we should like to see even a partial list of the remedies to which during his life our Connecticut martyr has probably resorted. He has carried a roll of brimstone in his left pantaloons pocket. He has carried a bit of magnetized iron in his right ditto. He has floated (so to speak) in Pain Killer. He has put his trust in "cam fire," and likewise in capsicum. Whatsoever things are hot, or bracing, or tonic, or rubefacient, he has resorted to—to the "King of Pain," to the "Ready and Rapturous Relief," to Oils, to Ointments, to Poor Men's Porous Plasters, to Red Flannel Shirts, to Galvanic Braces, to "Opydildock," to Herbs and Roots and Seeds—to all things which grow or flow, which are digged from the bowels of the earth, which are extorted from crucibles, which drip from retorts, which are rosy or pale in apothecary's bottles—to powders, to tinctures, to decoctions, to pills, to essences, to panaceas and to elixirs—to boluses and globules and infinitesimal dilutions—to hot rum and water, to cold gin and sugar, to brandy plain—to sweats and to starvation, to flesh diet and to fish diet and to fowl diet—and all in vain. After his forty years of fortitude his painful and adhesive ailment still remains; his "rheumatiz" sticks tighter than a brother.

